# Tumor-derived hepatocyte growth factor is associated with poor prognosis of patients with glioma and influences the chemosensitivity of glioma cell line to cisplatin in vitro

**DOI:** 10.1186/1477-7819-10-128

**Published:** 2012-06-28

**Authors:** You-feng Guo, Xiao-bing Wang, Xiao-ying Tian, Yang Li, Bin Li, Quan Huang, Meng Zhang, Zhi Li

**Affiliations:** 1 Department of Pathology, The First Affiliated Hospital, Sun Yat-sen University, 58 Zhongshan Road II, Guangzhou 510080, China; 2 Department of Pathology, Guangdong General Hospital, 106 Zhongshan Road II, Guangzhou 510080, China; 3 School of Chinese Medicine, Hong Kong Baptist University, 7, Baptist University Road, Kowloon Tong, Hong Kong, China; 4 Department of Neurosurgery, The First Affiliated Hospital, Sun Yat-sen University, 58, Zhongshan Road II, Guangzhou 510080, China

**Keywords:** Glioma, Hepatocyte growth factor (HGF), Proliferation, Chemosensitivity, Prognosis

## Abstract

**Background:**

We examined the association of tumor-derived hepatocyte growth factor (HGF) with the clinicopathological features of gliomas and investigated the effect of HGF inhibition on the biological behavior of tumor cells in vitro in order to determine whether HGF is a valuable prognostic predictor for glioma patients.

**Methods:**

Seventy-six cases of glioma were collected. The tumor-derived HGF expression, cell proliferation index (PI) and intratumoral microvessels were evaluated by immunohistochemistry. Correlation between immunostaining and clinicopathological parameters, as well as the follow-up data of patients, was analyzed statistically. U87MG glioma cells were transfected with short interference (si)-RNA for HGF, and the cell viability, migratory ability and chemosensitivity to cisplatin were evaluated in vitro.

**Results:**

Both high HGF expression in tumor cells (59.2%, 45/76) and high PI were significantly associated with high-grade glioma and increased microvessels in tumors (*P* < 0.05). However, only histological grading (*P* = 0.004) and high-expression of HGF (*P* = 0.008) emerged as independent prognostic factors for the overall survival of glioma patients. The tumor-derived HGF mRNA and protein expressions were significantly decreased in vitro after transfection of HGF siRNA. HGF siRNA inhibited the cell growth and reduced cell migratory ability. Moreover, HGF siRNA transfection enhanced the chemosensitivity of U87MG glioma cells to cisplatin.

**Conclusion:**

This study indicated that there was significant correlation among tumor cell-derived HGF, cell proliferation and microvessel proliferation in gliomas. HGF might influence tumor progression by modulating the cell growth, migration and chemoresistance to drugs. Increased expression of HGF may be a valuable predictor for prognostic evaluation of glioma patients.

## Background

Glioma is the most common type of primary brain tumor with the worst prognosis in humans [1,2]. Increasing evidence indicates that the rate of tumor cell proliferation, invasion and induction of tumor angiogenesis might be responsible for glioma progression. In contrast to other solid tumors of the body, gliomas are characterized by an infiltration into surrounding brain parenchyma as individual cells along anatomic structures [3]. This might be the reason why recurrence of gliomas frequently occurs within a 2-cm margin of the primary mass after surgical resection. Recent studies have demonstrated that the biological behavior of gliomas is associated with tumor cell migration ability, increased resistance to apoptosis, and decreased sensitivity to chemotherapy or radiotherapy [4-6]. However, the mechanisms involved in these processes remain to be validated.

Hepatocyte growth factor (HGF) is a multifunctional cytokine produced by both stromal and parenchymal cells that stimulates the motility and invasion of several cancer cell types, and induces angiogenesis [7]. HGF plays an important role in tumor progression and is associated with the prognosis of various human malignancies, including carcinoma of the stomach, liver, lung and nasopharynx [8-10]. In human gliomas, the expression of HGF and its receptor, c-Met, is associated with tumor grade [11]. Gene transfer of HGF to glioma cells enhances their tumorigenicity, tumor growth and tumor-associated angiogenesis [12]. Inhibition of HGF or c-Met expression leads to suppression of in vivo tumor formation and growth [13]. A recent study has also indicated that HGF levels in cerebrospinal fluid could be of prognostic value for predicting the mortality and recurrence of glioblastoma [14]. At the cellular level, the HGF and c-Met signaling pathway has been demonstrated to influence tumor formation and malignant progression by inducing cell cycle progression, tumor cell migration, invasion and tumor angiogenesis in various brain tumor cell lines, including those derived from glioma, medulloblastoma and neuroblastoma [15-17]. Moreover, HGF can affect tumor malignancy by inhibiting basal and radio-/chemotherapy-induced tumor cell death and apoptosis. A few studies have shown that HGF protects glioblastoma cells from DNA-damaging agents by activating PI3K/AKT anti-apoptotic pathways [13,18].

In the clinical setting, malignant gliomas are poorly sensitive to anti-proliferative drugs, and less than 30% of malignant gliomas respond to adjuvant chemotherapy. Chemoresistance in gliomas is based on a complex network of multiple pathophysiological mechanisms [19]. Although various molecular markers involved in chemotherapy resistance have been investigated, their direct roles in the chemosensitivity and prognostic value of gliomas, except MGMT (O^6^-methylguanine DNA methyltransferase), remain controversial [20]. Recently, a study has also exhibited that inhibition of c-Met enhanced the chemosensitivity of glioma cell lines to cisplatin, but no clear molecular mechanism involvement has emerged [21]. The relationship, if any, between HGF and chemoresistance in gliomas needs to be verified.

The aim of this study is to evaluate whether tumor-derived HGF acts as a potent predictive factor for tumor recurrence and prognosis for patients with gliomas, and whether HGF indeed affects tumor progression by altering the biological behavior of tumor cells and increasing drug resistance.

## Methods

### Specimens of gliomas

This study was carried out at the First Affiliated Hospital of Sun Yat-sen University and the Guangdong General Hospital (Guangzhou, China). Archival formalin-fixed, paraffin-embedded specimens from 76 Chinese patients who underwent surgery from 2001 to 2009 were recruited. All patients had intracranial gliomas and no history of other malignancies. Histological sections of the primary resected surgical specimens were reviewed by authoritative pathologists according to the criteria of the WHO histological classification [22]. All experimental protocols were carried out with the approval of the Committee on Use of Human & Animal Subjects in Teaching and Research of Sun Yat-sen University according to the Helsinki Declaration.

The patients were 52 males and 24 females with a median age of 47 years (range 8–76). Of these, 41 patients had high-grade gliomas, WHO grade III − IV, including 23 glioblastoma multiformes, 15 anaplastic astrocytomas and 3 anaplastic oligoastrocytomas. The other 35 had low-grade gliomas, WHO grade I–II, including 21 fibrillary astrocytomas, 5 pilocytic astrocytomas, 5 oligodendrogliomas, 3 ependymomas and 1 pleomorphic xanthoastrocytoma. Since gliomas may present as ill-defined lesions, various magnetic resonance imaging (MRI) sequence combinations do not provide a unique contour for tumor delineation. The extension of surgical resection was conducted by preoperative imaging. For presumed low-grade gliomas, manual segmentation was performed with region of interest analysis to measure tumor volumes (cm^3^) on the basis of FLAIR or T2 axial slices. For high-grade gliomas, a similar segmentation was made using the volume of contrast-enhancing tissue seen on T1-weighted MRI. After surgery, patients were followed up for a mean of 25.6 months (range, 3–58 months). None of the patients had received chemotherapy or radiotherapy before surgery.

After surgery, patients with high-grade gliomas, including glioblastoma multiforme and anaplastic astrocytoma, underwent conventional external-beam radiotherapy with a total dose of 60 Gy and continuous daily temozolomide (75 mg per m^2^ of body-surface area per day, 7 days per week from the first to the last day of radiotherapy), followed by six cycles of adjuvant temozolomide (150–200 mg per m^2^ for 5 days during each 28-day cycle). The patients with astrocytoma (WHO grade II) were treated with temozolomide 1 month after the initial surgery only. The patients with oligodendrogliomas and oligoastrocytomas underwent PCV chemotherapy [procarbazine, methyl-1-(2-chloroethyl)-1-nitrosourea (CCNU), and vincristine] every 6 weeks (42-day cycles) for two to five cycles [23-25].

### Immunohistochemistry and scoring

The sections were subjected to immunostaining using a ChemMate Envision/HRP Kit (DAKO, Denmark). Slides were deparaffinized in xylene, rehydrated in decreasing concentrations of ethanol and rinsed in phosphate-buffered saline. After blocking with normal serum for 10 min, the slides were incubated with a 1:100 dilution of rabbit polyclonal HGF antibody, a 1:500 dilution of the mouse monoclonal ki-67 antibody or a 1:100 dilution of mouse monoclonal CD34 antibody (Santa Cruz Biotechnology, Santa Cruz, CA, USA) for 60 min, respectively. Slides were detected by ChemMate Envision/HRP Kit for 30 min at room temperature, followed by developing with diaminobenzidine (DAB) for visualization. Negative controls were provided by substituting non-immune serum for the primary antibodies. The immunostaining results were evaluated and scored semiquantitatively by two pathologists without knowledge of the clinical data of patients. Evaluation of HGF expression was calculated by a double scoring system (stain intensity times stain area) as previously described [26]. Stain intensity was scored as 0 for no staining, 1 for weak staining, 2 for moderate staining and 3 for strong staining. The staining area was scored as 1 for less than 35%, 2 for 35–75% and 3 for >75% of tumor cells. High expression of HGF was defined when the immunostaining score was ≥ 4, whereas low expression of proteins was defined as a score <4. The proliferation index (PI) of gliomas was determined according to a previous description. Briefly, the percentage of ki-67-positive nuclei was counted in ten high-power fields in the areas with the highest density of labeled nuclei. The PI of each sample was the mean of the independent percentage of ki-67-positive signals by two observers. The high PI was defined as a value greater than 5%, whereas low PI of tumor was defined as a value ≤5% [22]. The counting of microvessels in gliomas was evaluated by the previously reported method [27]. In brief, intratumoral microvessel density (IMD) was observed in areas of the most intense neovascularization or hotspots in the tumor by light microscopy. After the area of the highest neovascularization had been determined, single microvessels were manually counted on a × 200 field. Any brown-stained endothelial cell or cell cluster that was clearly separated from the adjacent microvessels was considered as a single, countable microvessel, and the IMD value of each sample was the mean of the independent microvessels counted by two observers.

### Cell culture and reagents

In this study, the human U87MG cell line (HGF high-expressed glioma cell line [28]) was used. U87MG cells were maintained in Dulbecco’s modified Eagle’s medium (DMEM) with 10% heat-inactivated fetal calf serum (FCS) and antibiotics (50 U/ml penicillin and 100 mg/ml streptomycin, Gibco/Invitrogen) at 37°C in a humidified incubator with 5% CO_2_.

### Transfection of glioma cells with small interfering RNA (siRNA) targeting HGF

Human HGF siRNA (siHGF) and nontargeting siRNA control (siControl) were designed and synthesized by Ribo Biotechnology Co. (Beijing, China). The sequence was identified to be specific to the human HGF gene by using the BLAST search of the NCBI database. siControl was used for negative control purposes. siRNAs were reconstituted, and subsequent transfections were conducted in six-well plates using TurboFect siRNA Transfection reagent (Fermentas, USA), according to the manufacturer’s instructions. In brief, U87MG cells were seeded into six-well plates with a density of 2 × 10^5^ cells/well. Once the cells reached 80% confluence, they were treated with either siHGF or siControl (50 nM) complexed with TurboFect according to the manufacturer’s instructions. Ten percent FCS was added 4 h after transfection, and fresh DMEM with 10% FBS was added as needed after 24 h. Cells were collected at 48 h after transfection to assess HGF protein levels by Western blot and the other functional assays listed in the following sections.

### Polymerase chain reaction (PCR) for the detection of HGF mRNA expression levels

U87MG cells were transfected for 48 h, and total RNA was isolated from cells using Trizol reagent (Sigma-Aldrich, USA) according to the manufacturer’s instructions. First-strand cDNA (20 ul) was synthesized from 2 ug total RNA using oligo (dT) primers. An aliquot (2 ul) of cDNA was used as template for PCR amplification with primers specific for HGF (sense primer: 5´CCACACGAACACAGCTATCGGGG -3’; antisense primer: 5´-TGGGAGCAGTAGCCAACTCGGA-3´, Invitrogen, USA). PCR was performed in an automatic thermal cycler (Perkin-Elmer-Cetus, Norwalk, CT). Samples were amplified through 35 consecutive cycles with annealing temperature of 57°C. A 10-ul volume of each PCR product was analyzed by electrophoresis on 1.5% agarose gel containing 0.5 ug/ml ethidium bromide, and the bands were visualized under ultraviolet light.

### Western blotting assay

Transfected U87MG cells were lysed as described previously [29]. Equal protein samples were subjected to 12% SDS-PAGE electrophoresis, followed by the transfer to a polyvinylidene fluoride (PVDF) membrane, blocking in 5% fat-free milk and incubation with HGF (at 1:500 dilution) or GADPH antibody (Abcam, USA) at 4°C overnight. Detection was performed using horseradish peroxidase-conjugated secondary antibody and enhanced chemiluminescence reagents from Amersham (Amersham Life Sciences, UK). The relative optical density (ROD, ratio to GADPH) of each blot band was quantified by NIH Image software (Image J 1.36b).

### Immunofluorescence staining

Transfected U87MG cells were plated on coverslips, fixed with 4% paraformaldehyde/phosphate-buffered saline (PBS) for 15 min and permeabilized with 0.1% Triton X-100 in PBS for 2 min, and then incubated in PBS containing 5% skim milk for 1 h at room temperature. Cells were incubated with anti-HGF antibody (1:100, Abcam, USA) for 1 h at room temperature, followed by incubation with goat anti-rabbit IgG/Cy3 (1:500, Invitrogen, USA) for 1 h and nuclear counterstaining with DAPI.

### MTT assay

The effect of siHGF on the viability of U87MG glioma cells was measured by 3-[4, 5-dimethylthiazol-2-thiazolyl]-2, 5-diphenyltetrazolium bromide (MTT) assay as described previously [30]. Briefly, U87MG cells were seeded at 1 × 10^5^ cells/well into 96-well plates in quintuplicate, and transfected with siHGF or siControl for 24, 48 and 72 h, respectively. Four hours before the desired time points, 20 ul of 5 mg/ml MTT was added into each sample. The plates were then incubated at 37°C in a 5% CO_2_/95% air atmosphere for 4 h. Thereafter, the medium was discarded, and 150 ul of DMSO was added into each well. The absorbance was determined at 490 nm by an enzyme-linked immunosorbent assay reader. Results represented the OD ratio between the siRNA-treated and untreated cells at the same indicated time points.

### In vitro wound healing assay

Migratory ability of U87MG cell was measured using the in vitro wound healing assay. Cells were seeded in six-well plates and transfected with siHGF or siControl for 48 h. Transfected cells were grown to 100% confluence. Wounds were created by scraping monolayer cells with a sterile pipette tip. At 0, 12, 24 and 48 h after the creation of wounds, wound distances were measured at each time point and expressed as the average percent of wound closure by comparing the zero time.

### Cisplatin cytotoxicity assay

For the acute cytotoxicity assay, U87MG cells were seeded at 1 × 10^4^ cells per well in 96-well plates, and were transfected by siHGF or siControl for 48 h, and subsequently exposed to cisplatin at final concentrations of 0.5, 1.0, 2.0, 4.0 or 8.0 ug/ml for 24 h in triplicate wells. Cell survival was determined using a previously described colorimetric MTT assay. Then 20 ul of 5 mg/ml MTT was added to each well, and the plates were incubated for 4 h at 37°C. The absorption was read at 490 nm using an automated microplate reader. Mean cell viability was calculated by the ratio of absorbance units of transfected cell samples to the mean absorbance units of the control cell samples. All the experiments were repeated at least three times. The IC50 value is defined as concentration of cisplatin that is required for a 50% reduction in absorbance calculated from the growth curves.

### Statistical analysis

All statistical analysis was carried out by SPSS 13.0 software for Windows. The chi-square test was used to assess HGF expression and PI with clinicopathological characteristics. Vascular density and data derived from experiments in vitro were given as the mean ± SD as indicated. Data were analyzed by one-way ANOVA with Dunnett’s post hoc test and Tukey’s post hoc test for multigroup comparisons. The survival curve of patients was determined by the Kaplan-Meier method and Cox regression, and statistical evaluation was performed using the log rank test. A value of *P* < 0.05 was considered statistically significant.

## Results and discussion

### Correlation among HGF expression, cell proliferation and microvessel counts in gliomas

**Figure 1:**
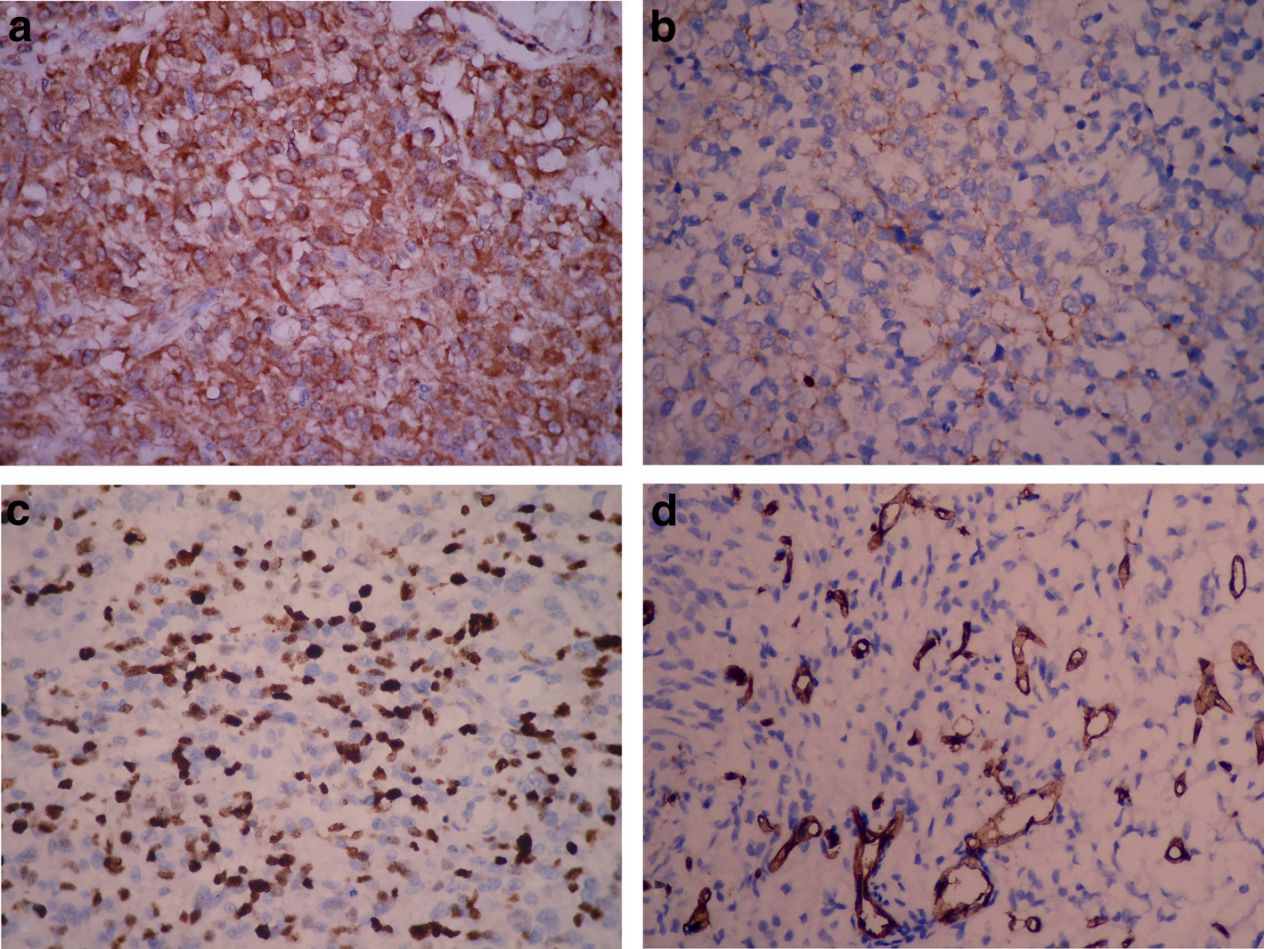
**Immunohistochemical staining of glioma tissues. a** Strong and diffuse expression of HGF was found in high-grade glioma; **b** positive staining of HGF was shown focally and weakly in low-grade gliomas. **c** The extent of Ki-67-positive signal represented the proliferation of gliomas. Most high-grade gliomas had a high proliferation index (*PI*). **d** Intratumoral microvessels of gliomas were highlighted by staining endothelial cells for anti-CD34. Any brown-staining endothelial cell or cell cluster that was clearly separated from the adjacent microvessels was considered as a single, countable microvessel. (**a**-**c** immunohistochemical staining with original magnification, ×400; **d** immunohistochemical staining × 200).

Positive cytoplasmic HGF staining was observed in tumor cells of gliomas in various degrees (Figures 1a, b). High HGF expression in tumor cells was observed in 59.2% (45/76) of glioma tissues. High HGF expression was significantly associated with higher histological grading (WHO grades III and IV) and tumor recurrence (*P* = 0.001). Nuclear-positive signals with intense dark brown staining for proliferative tumor cells were observed in gliomas labeled by ki-67 antibody (Figure 1c). The mean proliferation index (PI) values were significantly higher in cases with high HGF expression than those in cases with low expression of HGF (*P* = 0.001). Higher PI values were more easily observed in the cases with higher histological grading (*P* = 0.001). However, the PI value showed no correlation with other clinicopathological parameters, including tumor recurrence and microvessel counts in tumors. Microvessels in gliomas, specifically stained by anti-CD34 immunostaining, were observed in all specimens and scored as intratumoral microvessel density (IMD) (Figure 1d). The mean IMD value was 22.4/HPF, but with great individual variation (range 10–50). The IMD was significantly higher in tumors with high expression of HGF or with high-grade gliomas. However, there was no significant correlation found between the IMD value and tumor recurrence (Table 1).

**Table 1:** Correlation among HGF expression, cell proliferation, IMD value and clinicopathological parameters of patients with gliomas

**Variable**	**HGF expression**^**a**^		**Cell proliferation**^**a**^		**IMD value**^**b**^**(mean ± SD)**
	**Low exp. (*n* = 31)**	**High exp. (*n* = 45)**	**Low PI (*n* = 33)**	**High PI (*n* = 43)**	
Histological grade					
High grade (*n* = 41)	10	31	3	38	24.78 ± 10.30
Low grade (*n* = 35)	21	14	30	52	19.57 ± 8.62
	*P* = 0.001		*P* = 0.001		*P* = 0.021
Tumor recurrence					
No (*n* = 57)	27	30	26	31	21.70 ± 9.83
Yes (*n* = 19)	4	15	76	12	24.42 ± 9.89
	*P* = 0.002		*P* = 0.323		*P* = 0.301
HGF expression					
Low expression (*n* = 31)			24	7	18.24 ± 6.41
High expression (*n* = 45)			9	36	25.23 ± 10.82
			*P* = 0.001		*P* = 0.001
Cell proliferation					
Low PI (*n* = 33)	24	9			19.31 ± 8.32
High PI (*n* = 43)	7	36			24.73 ± 10.37
	*P* = 0.001				*P* = 0.016

^a^Chi-square test.

^b^Independent sample *T* test.

### Association of HGF expression with survival of patients with gliomas

**Figure 2:**
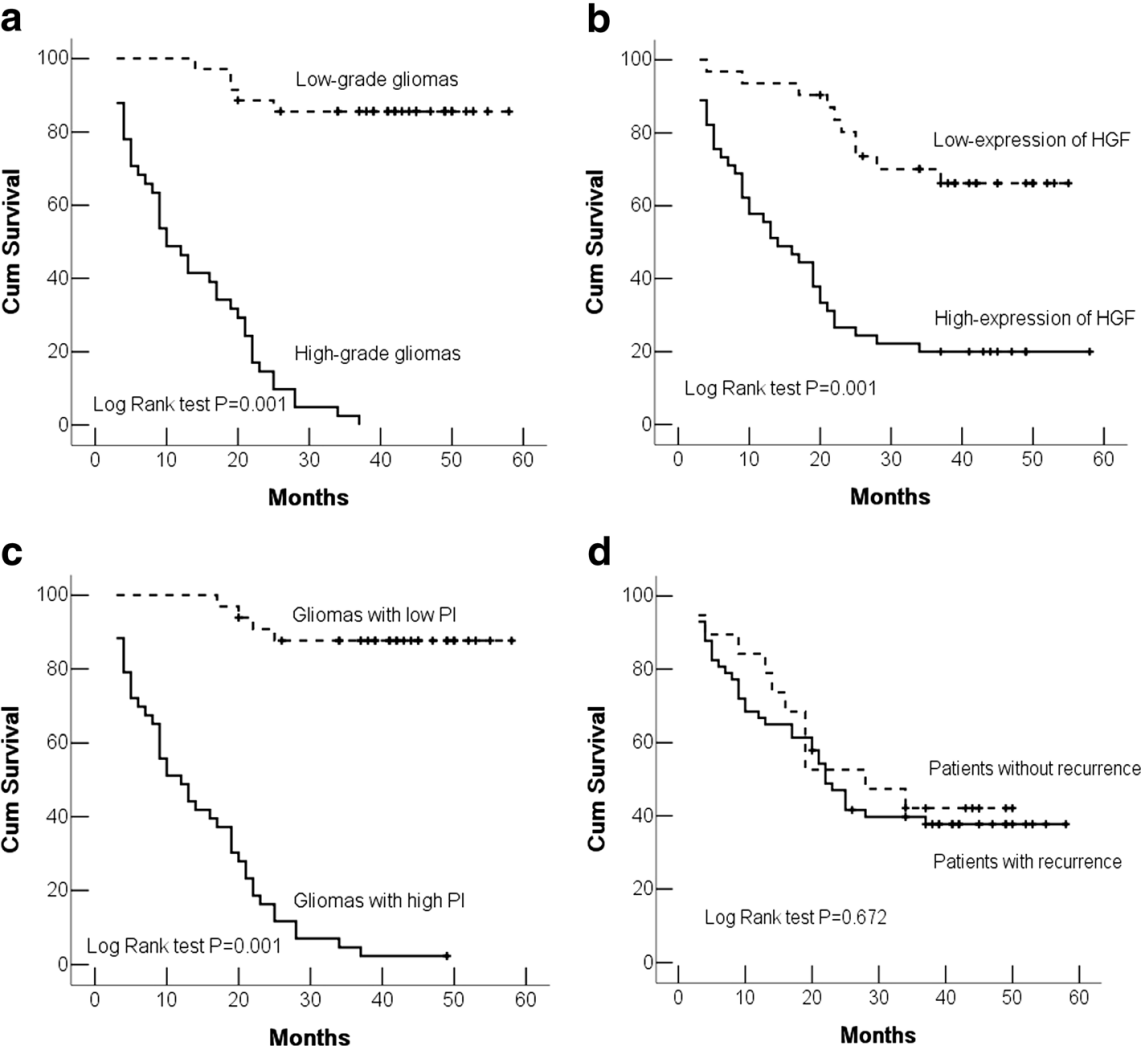
**Kaplan-Meier survival analysis in 76 patients with gliomas. a** Kaplan-Meier curve showing the patients with high-grade tumor have a lower survival rate than those with low-grade tumor. **b** Patients with a tumor with high HGF expression had a lower survival rates than those with low HGF expression tumors. **c** A significant difference in survival rates was found between patients with a high proliferation index and low index in their tumor. **d** There was no significant difference in survival rates between the patients with or without tumor recurrence.

The 76 patients were followed up from 3 to 58 months with a mean period of 25.6 months, and 46 (60.5%) died of their tumor during this period. In univariate analysis, high-grade tumor, high-expression of HGF and higher PI were significantly associated with a short survival time of patients with gliomas. There was no significant difference in survival time between the patients with or without tumor recurrence (Figure 2, Table 2). However, in multivariate analysis, only histological grade and high expression of HGF in gliomas were independently associated with survival. Other clinical parameters, such as age, gender, microvessels, cell proliferation and tumor recurrence, showed no association with patient survival (Table 3).

**Table 2:** Kaplan-Meier analysis for overall survival rate of patients with gliomas

**Characteristics**	**Mean survival time (months)**	**95% confidence interval (months)**	*P *values*
Age (years)			
<47 (*n* = 37)	33.98 ± 3.67	26-41	0.324
≥47 (*n* = 39)	26.63 ± 3.16	20-32	
Gender			
Male (*n* = 52)	31.46 ± 3.09	25-37	0.985
Female (*n* = 24)	25.92 ± 3.41	19-32	
Histological grade			
High grade (WHO III-IV) (*n* = 41)	13.61 ± 1.47	10-16	0.001
Low grade (WHO I-II) (*n* = 35)	52.45 ± 2.31	47-56	
Tumor recurrence			
No (*n* = 57)	30.49 ± 3.01	24-36	0.672
Yes (*n* = 19)	30.42 ± 4.13	22-38	
HGF expression			
High expression (*n* = 45)	21.46 ± 2.93	15-27	0.001
Low expression (*n* =31)	43.67 ± 3.04	37-49	
Cell proliferation			
High PI (*n* = 43)	14.41 ± 1.60	11-17	0.001
Low PI (*n* = 33)	53.44 ± 2.13	49-57	
Intratumoral microvessel density			
≥ Mean IMD value (*n* = 27)	24.46 ± 4.05	16-32	0.079
< Mean IMD value (*n* = 49)	34.55 ± 3.13	28-40	

*Log rank test.

**Table 3:** Cox regression model for multivariate analyses of prognostic factor in gliomas

**Variable**	**Hazard ratio**	**95% confidence interval**	**P value**
Age (<47 vs. ≥ 47)	0.924	0.446-1.914	0.833
Gender (male vs. female)	1.144	0.534-2.452	0.728
Histological grade (WHO I-II vs. WHO III-IV)	7.282	1.849-28.671	0.004
Tumor recurrence (no vs. yes)	0.615	0.269-1.403	0.248
Cell proliferation (low PI vs. high PI)	3.898	0.848-17.913	0.080
Angiogenesis (IMD value)	0.992	0.961-1.025	0.666
HGF expression (low vs. high)	3.327	1.357-8.152	0.008

### Effect of HGF siRNA on HGF expression in glioma cells in vitro

**Figure 3:**
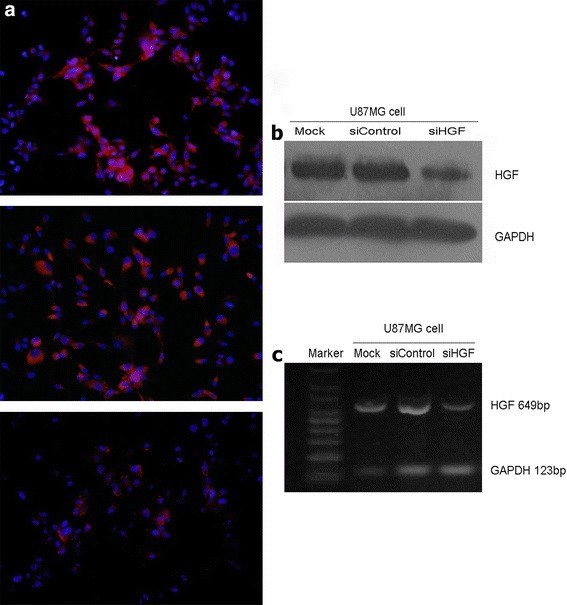
**HGF siRNA inhibited tumor cell-derived HGF expression. a** Immunofluorescence assay showed that tumor cell-derived HGF protein expression was significantly decreased in U87MG glioma cells with HGF siRNA transfection (*bottom*) when compared with control siRNA transfected cells (*middle*) and mock (*top*) (immunofluorescence staining with original magnification, ×400). Western blot assay (**b**) and RT-PCR (**c**) exhibited that HGF protein and mRNA expression levels were decreased in the cells with HGF siRNA transfection.

U87MG glioma cells were transfected with siHGF and siControl for a period of 48 h. The transfected cells were highlighted by red dot-like fluorescence under the fluorescence microscopy. The ratio of transfection in U87MG glioma cell was 50%. We investigated the status of HGF in glioma cells by immunofluorescence staining, Western blotting and RT-PCR assay. By immunofluorescence test and Western blotting assay, the HGF protein level was significantly decreased in U87MG cells with siHGF treatment compared to those with siControl transfection and untreated cells (Figure 3a and b). By RT-PCR assay, we found the HGF mRNA level was also dramatically decreased in the cell after 48 h of siHGF transfection (Figure 3c).

### Effect of HGF siRNA on growth inhibition and migratory ability of glioma cells in vitro

**Figure 4:**
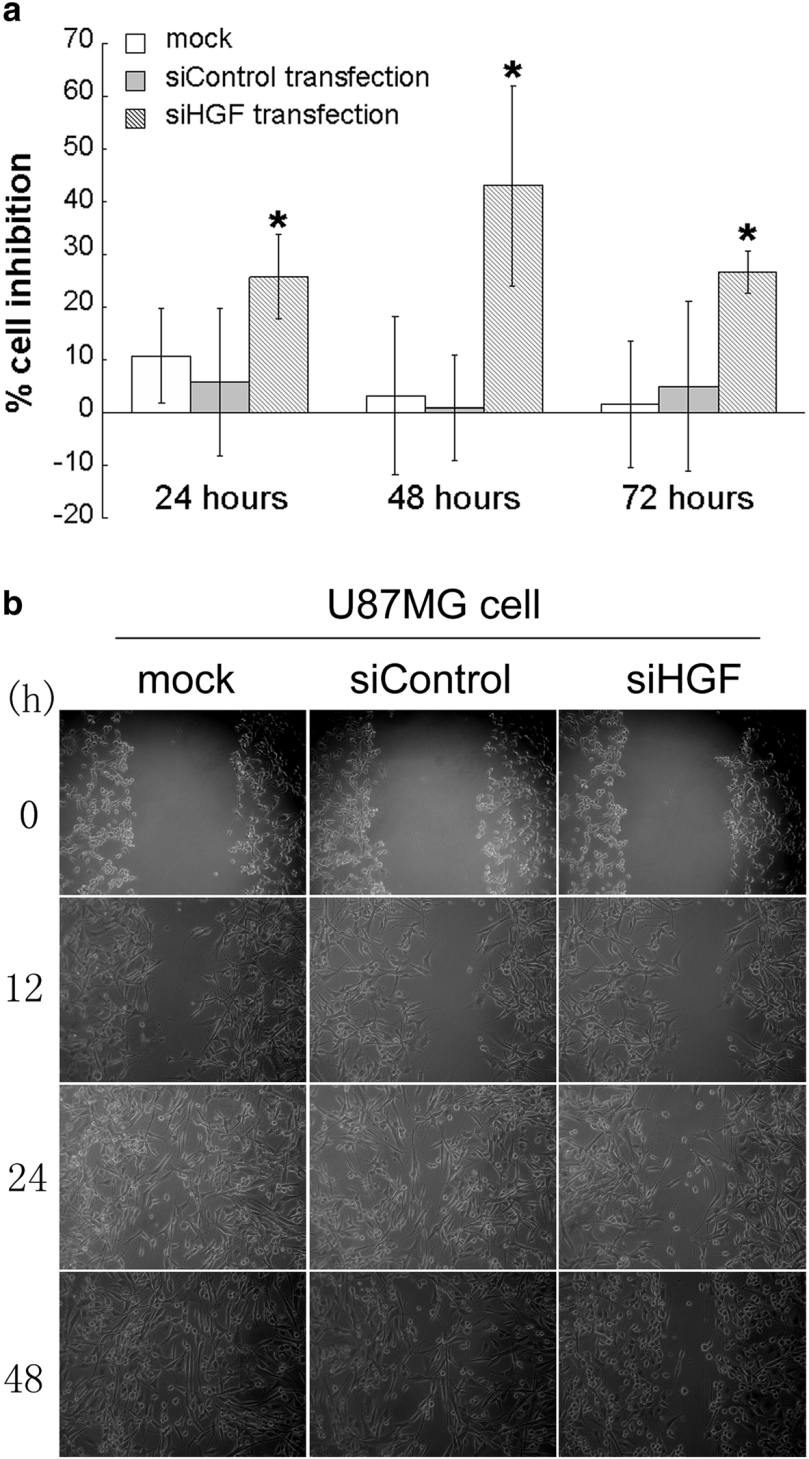
**The effect of HGF inhibition on cell growth and migratory abilities of glioma cells. a** MTT assays were performed in the U87MG glioma cell line. Cells were cultured in a 96-well plate and transfected with HGF siRNA or control siRNA for 24, 48 and 72 h points with an average of five independent experiments in each group (*P <0.05 versus control cells; one-way ANOVA). **b** Optical microscopic images of in vitro wound healing at 0, 12, 24 and 48 h after the creation of wounds. HGF siRNA-transfected cells displayed significantly slower wound closure at all time points compared with control siRNA-transfected cells.

The effect of HGF siHGF on cell viability was evaluated by MTT assay. As shown in Figure 4a, siHGF transfection resulted in inhibition of glioma cell viability. The inhibitory effect of siHGF on glioma cell growth was strongest after 48 h of transfection. There was a significant difference in cell growth inhibition between the HGF siRNA-transfected cells and the control cells (*P* <0.05). Wound healing assay showed that the migration in HGF siRNA-transfected cells was markedly decreased compared with those in control siRNA-transfected and untreated cells (Figure 4b).

### siHGF-induced chemosensitivity enhancement to cisplatin in glioma cells in vitro

**Figure 5:**
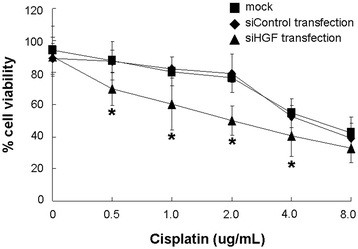
**HGF siRNA-induced enhancement of chemosensitivity to cisplatin. Sensitivity curve of the U87MG cell line toward cisplatin via MTT assay.** Growth inhibition of HGF siRNA-transfected cells was observed after exposure to different concentrations of cisplatin for 24 h (*P < 0.05 versus control cells, one-way ANOVA).

We used a cytotoxicity MTT assay to further investigate the effect of HGF siRNA on the chemosensitivity to cisplatin in glioma cells. Exposure of HGF siRNA-transfected U87MG cells to different concentrations of cisplatin induced significant proliferative inhibition. When the concentration of cisplatin was 8 ug/ml; the viability of HGF siRNA-transfected U87MG cells showed no significant difference compared with those of the siControl-transfected and untreated cells (Figure 5). Meanwhile, the IC50 concentration of cisplatin for U87MG cells decreased significantly from 7.06 ug/ml in control cells and 2.01 ug/ml in siHGF-transfected cells, respectively, which indicated that siHGF might be one of the factors that enhanced the chemosensitivity of glioma cells to cisplatin.

As a multifunctional cytokine, HGF and its receptor tyrosine kinase, c-Met, have emerged as key determinants of tumor development and progression, including brain tumors [31]. The extent of HGF expression as a prognostic factor has also been demonstrated in hepatocellular carcinoma, gastric cancer and breast cancer [9,32,33]. In gliomas, HGF-associated tumor growth and angiogenesis have been demonstrated. The HGF expression level could be of prognostic value for predicting the mortality and recurrence of tumors. This study showed that high levels of HGF expression were found in glioma specimens with higher histological grade and tumor recurrence, and these were closely associated with shorter patient survival in both univariate and multivariate analysis. These results corroborate previous findings that suggest HGF as a valuable prognostic factor in patients with gliomas.

Previous studies have indicated that HGF-induced cell proliferation and anti-apoptosis are involved in the progression of tumors [28,31]. Moreover, accumulated evidence revealed that HGF might be identified as the most potent stimulator of glioma cell migration when compared with numerous other growth factors previously associated with glioma motility [34]. In this study, we found that the reduction of tumor-derived HGF expression resulted in inhibition of cell proliferation and impaired the ability of cell migration in vitro. We postulated that tumor cell-derived HGF was likely to influence the prognosis of glioma patients by way of an autocrine regulatory loop [17,35]. In the clinical setting, measurement of the HGF level in tumor cells might be used as a predictive element directly associated with the degree of malignancy and the hazard rating of recurrence of gliomas. In addition, there is some debate over aggressive therapy for low-grade gliomas because neither histopathological nor clinical data are currently taken as reliable recurrence predictors for low-grade gliomas, especially for grade I tumors. However, in this study, we found that all pilocytic astrocytomas (WHO grade I) with higher HGF expression recurred during the follow-up period despite the low level of proliferative activity (data not shown). Although the number of grade I gliomas examined in this study was not sufficient to allow us to draw a definite conclusion, these findings indicated that high expression of HGF could be used as a predictor for the recurrence of gliomas and could help determine whether aggressive therapy is necessary, particularly for those gliomas with lower WHO grades.

Analysis of a wide range of gliomas showed a gross correlation of cell proliferation with histological grade of tumor. The proliferation index, as determined by the antibody Ki-67/MIB-1, however, showed great regional variation within a tumor, and might overlap with values for low- and high-grade gliomas. Despite the higher proliferation indices observed in high-grade gliomas, an association between proliferation index and clinical outcome has not been demonstrated [36]. HGF-induced in vitro proliferation and anchorage-independent growth have been demonstrated in various brain tumor cell lines [15-17] in which HGF plays a mitogenic role in tumor cells at least partly by mediating the G1/S cell cycle transition. In this study, we found tumor-derived HGF was closely associated with cell proliferation in both in vivo and in vitro investigations. However, multivariate analysis for overall survival in patients indicated that cell proliferation in tumors was not an independent predictive factor for the prognosis of gliomas, although high PI was significantly correlated with a high histological grade and a decreased survival rate in patients. In contrast to our results, however, several studies have shown that the cell proliferation potential was independently correlated with outcome in intracranial gliomas [37,38]. Further investigations with larger case numbers and unabridged follow-up data should be carried out to clarify whether the detection methods and sample size are responsible for these discrepancies.

Massive formation of blood vessels is associated with a high histological grade, which is unfavorable for the outcome of gliomas. Significant evidence exists showing that HGF promotes the angiogenesis of brain tumors. HGF could be detected in the tumor blood vessels and induce tumor endothelial cell proliferation and migration by paracrine and autocrine mechanisms [39]. In addition to its direct angiogenic activities, HGF could enhance the induction of angiogenic factor in gliomas, including VEGF, bFGF and IL-8. In the present study, we found that microvascular proliferation in gliomas was significantly correlated with high expression of tumor-derived HGF, as well as higher histological grade. However, multivariate analysis for overall survival in patients indicated that angiogenesis in tumors was not an independent predictive factor for the prognosis of gliomas. Although the expression of HGF in tumor endothelial cells and the expression of angiogenic factors were not analyzed in this study, it is reasonable to believe that tumor-derived HGF can stimulate tumor neovascularization at least partly by mediating the secretion of relevant angiogenic factors via a paracrine mechanism.

Gliomas are frequently resistant to therapy even after aggressive surgical resection, external beam radiation therapy and the maximum tolerated chemotherapy dose with agents such as temozolomide or nitrosourea. Enhancement of chemosensitivity is one of the major strategies to overcome the multidrug resistance and undesirable side effects of chemotherapy. The mechanism of chemoresistance in tumor therapy is very complicated and remains poorly understood. Several studies have demonstrated that inhibition of PI3K/AKT signaling and p44/42 MAPK activation is an efficient way to attenuate the resistance of chemotherapy [40,41]. It has been revealed that HGF-activated c-Met expression can protect glioblastoma cells and tumor xenografts from DNA-damaging agents just by activating the PI3K/AKT pathway [18]. In the present study, we found that inhibition of tumor-derived HGF by siRNA could dramatically enhance the sensitivity of tumor cells to cisplatin in vitro. This indicated that inhibited expression of HGF or interfering c-Met activation in gliomas appears to be a promising strategy for developing a therapeutic approach to treat this malignant tumor. Cisplatin alone or in combination with other chemotherapy agents has been used to treat low-grade gliomas or recurrent glioblastoma [42,43], because local chemotherapy of glioblastoma with cisplatin followed by irradiation proved to be well tolerated and effective [44]. However, cisplatin is not a first-line drug for glioma chemotherapy because of its limited ability to reach an effective concentration at the tumor site. Therefore, an HGF inhibitor combined with cisplatin might be a potential application to enhance the chemosensitivity of gliomas with higher levels of HGF.

## Conclusion

Although the precise factors responsible for a poor prognosis in gliomas have not been identified, this study indicates high expression of HGF in tumor cells may play a critical role in tumor progression and is a valuable predictor for prognosis evaluation in glioma patients. To the best of our knowledge, the present data provide a correlation between the HGF status in tumor cells and chemosensitivity to cisplatin in vitro for the first time. This might provide a new promising strategy for a therapeutic approach for patients with gliomas.

## Competing interests

The authors declare that they have no competing interests.

## Authors’ contributions

Y-FG and X-BW made contributions to the acquisition of clinical data and analysis of the histological features by H&E staining, and carried out the cellular studies. They are co-first authors and made equal contributions to this work. X-YT drafted the manuscript. BL and QH carried out the immunoassays. YL and MZ participated in the design of the study and performed the statistical analysis. ZL critically revised the manuscript for important intellectual content and gave final approval of the version to be published. All authors read and approved the final manuscript.
